# RTS,S/AS02A Malaria Vaccine Does Not Induce Parasite CSP T Cell Epitope Selection and Reduces Multiplicity of Infection

**DOI:** 10.1371/journal.pctr.0010005

**Published:** 2006-05-19

**Authors:** Sonia Enosse, Carlota Dobaño, Diana Quelhas, John J Aponte, Marc Lievens, Amanda Leach, Jahit Sacarlal, Brian Greenwood, Jessica Milman, Filip Dubovsky, Joe Cohen, Ricardo Thompson, W. Ripley Ballou, Pedro L Alonso, David J Conway, Colin J Sutherland

**Affiliations:** 1 Centro de Investigação em Saúde da Manhiça, Ministério de Saúde, Maputo, Mozambique; 2 Instituto Nacional de Saúde, Ministério de Saúde, Maputo, Mozambique; 3 Centre de Salut Internacional, Hospital Clínic/IDIBAPS, University of Barcelona, Barcelona, Spain; 4 GlaxoSmithKline Biologicals, Rixensart, Belgium; 5 Gates Malaria Partnership, London School of Hygiene and Tropical Medicine, London, United Kingdom; 6 PATH Malaria Vaccine Initiative, Bethesda, Maryland, United States; 7 Medical Research Council Laboratories, Fajara, Gambia; 8 HPA Malaria Reference Laboratory, London School of Hygiene and Tropical Medicine, London, United Kingdom

## Abstract

**Objective:**

The candidate malaria vaccine RTS,S/AS02A is a recombinant protein containing part of the circumsporozoite protein (CSP) sequence of *Plasmodium falciparum,* linked to the hepatitis B surface antigen and formulated in the proprietary adjuvant system AS02A. In a recent trial conducted in children younger than age five in southern Mozambique, the vaccine demonstrated significant and sustained efficacy against both infection and clinical disease. In a follow-up study to the main trial, breakthrough infections identified in the trial were examined to determine whether the distribution of *csp* sequences was affected by the vaccine and to measure the multiplicity of infecting parasite genotypes.

**Design:**

P. falciparum DNA from isolates collected during the trial was used for genotype studies.

**Setting:**

The main trial was carried out in the Manhiça district, Maputo province, Mozambique, between April 2003 and May 2004.

**Participants:**

Children from the two cohorts of the main trial provided parasite isolates as follows: children from Cohort 1 who were admitted to hospital with clinical malaria; children from Cohort 1 who were parasite-positive in a cross-sectional survey at study month 8.5; children from Cohort 2 identified as parasite-positive during follow-up by active detection of infection.

**Outcome:**

Divergence of DNA sequence encoding the CSP T cell–epitope region sequence from that of the vaccine sequence was measured in 521 isolates. The number of distinct P. falciparum genotypes was also determined.

**Results:**

We found no evidence that parasite genotypes from children in the RTS,S/AS02A arm were more divergent than those receiving control vaccines. For Cohort 1 (survey at study month 8.5) and Cohort 2, infections in the vaccine group contained significantly fewer genotypes than those in the control group, (*p =* 0.035, *p =* 0.006), respectively, for the two cohorts. This was not the case for children in Cohort 1 who were admitted to hospital (*p =* 0.478).

**Conclusions:**

RTS,S/AS02A did not select for genotypes encoding divergent T cell epitopes in the C-terminal region of CSP in this trial. In both cohorts, there was a modest reduction in the mean number of parasite genotypes harboured by vaccinated children compared with controls, but only among those with asymptomatic infections.

## Editorial Commentary

**Background:** A recent trial in young children in Mozambique found that an experimental malaria vaccine, RTS,S/AS02A, reduces the risk of clinical malaria, delays time to new infection, and reduces episodes of severe malaria over 18 months. RTS,S/AS02A is based on a particular protein, the circumsporozoite protein, which is expressed on the surface of the malaria parasite at a stage in its life cycle prior to the disease-causing blood-stage infection. Because there are different variants of the circumsporozoite protein in the parasite population, the researchers were concerned that the strain-specific protection offered by RTS,S/AS02A might select for parasites with particular variants of the circumsporozoite gene. They did a follow-up study to investigate this concern, looking to see whether vaccinated children who became infected with malaria had variant forms of the parasite.

**What this trial shows:** The investigators found that there was no increase in how different the variants were from the vaccine-type allele between vaccinated and control individuals. There was a small decrease in the average number of parasite “strains” in vaccinated children compared with controls. However, in children within the trial who were admitted to hospital with clinical malaria, the number of different “strains” of the parasite was no different between vaccinated children and controls.

**Strengths and limitations:** The study asks an important molecular question about whether vaccination with RTS,S/AS02A selects for different variants, specifically escape mutants of the malaria parasite. If the researchers had found such selection, this would suggest that the vaccine's success could be compromised once widely deployed. A limitation of this follow-up study is that it is difficult to interpret these findings because the mode of action of the vaccine is not yet fully understood. It is also difficult to understand how the results might apply to non-hospitalised patients with clinical malaria, because the malarial patients included in this study were exclusively identified through hospital admissions.

**Contribution to the evidence:** This study adds molecular information to the previously reported results on the clinical effectiveness of the RTS,S/AS02A vaccine. The researchers found no evidence that the RTS,S/AS02A vaccine selected for different variants of the malaria parasite.

## INTRODUCTION

Malaria continues to be a major cause of morbidity and mortality in many countries of Africa, and is estimated to cause more than one million deaths each year [1]. Whereas better use of available control measures and the search for new, cheap, and effective drugs are the main aims of current efforts in malaria research, sustainable control of malaria would be enhanced if effective vaccines were available for use in combination with other control measures.

Several malaria vaccine candidates have been tested in malaria endemic countries [2], including the pre-erythrocyte vaccine RTS,S/AS02A, a recombinant polypeptide developed by GSK Biologicals that contains most of the C-terminal half of the circumsporozoite protein (CSP) sequence of P. falciparum fused to hepatitis B virus surface antigen formulated in the AS02A adjuvant system [3,4]. Efficacy against P. falciparum infection in both experimentally infected volunteers and naturally exposed adults have been obtained with this vaccine [3–5], and the protective efficacy of RTS,S/AS02A against clinical malaria was demonstrated recently in a proof-of-concept Phase IIb, double-blind, randomised, controlled trial in children aged 1–4 years in southern Mozambique [6]. Vaccine efficacy for the first or only episode was 29.9% (95% CI 11.0–44.8; *p =* 0.004) for clinical malaria and 57.7% (95% CI 16.2–80.6; *p =* 0.019) for severe malaria over six months of follow-up. The vaccine efficacy for extending time to first P. falciparum infection was 45.0% (95% CI 31.4–55.9, *p* < 0.001). Extended follow-up has confirmed that protection is sustained over 18 months post-vaccination [7].

CSP is an abundant protein found on the surface of the sporozoite. The prevailing hypothesis in this field is that cell-mediated immune responses play an important role in protection against pre-erythrocytic parasites, and studies of the gene encoding CSP of P. falciparum have demonstrated a significant level of genetic polymorphism among isolates, the most variable domains being the T cell epitopes near the C-terminus of the protein, which are designated Th2R and Th3R [8,9]. The inclusion of such variable sequences in the vaccine may select for non vaccine-type alleles that could impact on overall efficacy, as non vaccine-type alleles were selected by strain-specific vaccine responses to P. falciparum merozoite protein MSP-2 among vaccinated Papua New Guinean children [10]. In adult Gambian males, the RTS,S/AS02A vaccine was found to induce protection against P. falciparum infection that was not strain-specific [11]. The parasite population in this study exhibited a high frequency of alleles encoding T cell epitopes with the same amino-acid sequence as those in the vaccine, which is based on laboratory P. falciparum clone 3D7/NF54. Of the *csp* alleles identified by dot-blotting, 10% encoded Th2R and 15% encoded Th3R epitopes that were indistinguishable from those in the vaccine. The prevalence of these alleles was similar among emergent infections in the vaccine and the control groups [11]. Interestingly, in the Gambian trial the number of distinct parasite genotypes per infection (multiplicity of infection (MOI)) in breakthrough cases was higher in the RTS,S/AS02A group than in the control group (4.90 versus 4.23, *p =* 0.05). These studies indicate that integrated molecular typing will be a useful tool for the evaluation of malaria vaccines, especially where a strain-specific effect is possible.

As part of the trial conducted in 2003–2004 by Alonso and colleagues to evaluate the efficacy of the RTS,S/AS02A vaccine in Mozambican children [6], DNA sequences encoding the Th2R and Th3R epitopes of the CSP gene of parasites from breakthrough cases in both vaccine and control arms of the trial were obtained during six months of follow-up. These were analysed to test the hypothesis that protection against infection or clinical disease following vaccination with RTS,S/AS02A was not sequence-dependent with regard to the Th2R and Th3R epitopes of CSP. In addition, we investigated whether vaccination modified the number of P. falciparum genotypes present in infected subjects.

## METHODS

### Participants

Samples for molecular analysis were collected during the course of a vaccine trial conducted in the Manhiça district, southern Mozambique, between April 2003 and May 2004. Details of the study area, population, and study design have been previously reported [6]. In brief, a Phase IIb, double-blind, randomised, controlled trial was conducted to determine the efficacy of the vaccine against clinical disease and infection in children aged 1–4 years. These endpoints were measured in two cohorts based at different sites, Cohorts 1 (Manhiça) and Cohort 2 (Ilha Josina). Malaria transmission intensity is higher in the study area of Cohort 2 than in Cohort 1 [6]. In Cohort 1, 1,605 children were enrolled and the primary endpoint was time to the first clinical episode of symptomatic P. falciparum malaria over a six-month observation period starting 14 d after dose 3, corresponding to study month 2.5, until study month 8.5. In Cohort 2, 417 children were enrolled and the main endpoint was the efficacy of the vaccine for prevention of new infections, over the post-vaccination follow-up period spanning study months 2.5 to 8.5. In Cohort 2, subjects received a single dose of sulfadoxine-pyrimethamine and a daily dose of amodiaquine for 3 d, 4 wk prior to the commencement of surveillance and 2 wk prior to the last dose of vaccine, to clear any parasitaemia.

**Figure 1:**
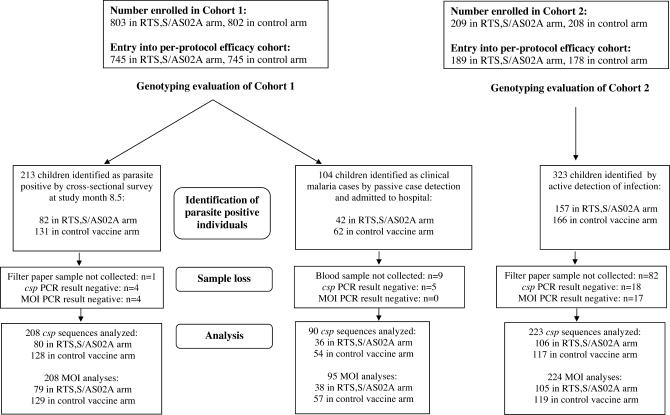
Participant Flow and Samples Analysed The diagram begins with the per-protocol efficacy cohort described in Figures 2 and 3 of [6].

Samples for parasite genotyping collected from children in Cohort 1 were obtained from two sources: 1) filter-paper bloodspots from a cross-sectional survey conducted at study month 8.5, and 2) frozen EDTA-blood from patients with malaria identified by passive case detection and admissions to the hospital as in-patients. In Cohort 2, filter-paper bloodspot samples were collected from patients with malaria infection identified by active detection of infection (see Figure 1). In both cohorts, only peripheral blood samples from those children who were fully compliant with the vaccination protocol were included.

The protocol was approved by the National Mozambican ethics review committee, the Hospital Clínic of Barcelona ethics review committee, the PATH human subjects protection committee, and the London School of Hygiene & Tropical Medicine ethics review committee. Written informed consent was obtained from parents/guardians prior to study enrollment and blood sample collection.

### Blood Sample Collection and DNA Extraction

Filter-paper blood samples were air dried, individually wrapped in aluminum foil, placed in a box containing desiccant (silica gel), and stored at 4 °C until use. All samples were labelled with a unique sample identification number.

DNA extraction from one spot (three fragments, approximately 3-mm diameter) of filter paper and from about 200 μl of frozen blood samples (hospitalised Cohort 1 participants only) was performed using the QIAamp DNA blood minikit-extraction procedure as described by the manufacturer (Qiagen, Valencia, California, United States). DNA samples were eluted into 150 μl of buffer AE and stored at −20 °C until use.

### Determination of *CSP* Polymorphic Sequences

The distribution of polymorphic sequence variants encoding the Th2R and Th3R regions of P. falciparum CSP was determined using a semi-nested PCR followed by sequencing of purified PCR products.

Amplification by semi-nested PCR of a 321-bp fragment of the *csp* gene was performed using three primers binding to conserved sequences flanking the Th2R and Th3R regions of *csp*. For the first round PCR, the primers were: forward primer (CSo101), 5′-aatcaaggtaatggacaagg-3′; reverse primer (CSo102), 5′-ctaattaaggaacaagaagg-3′. For the second reaction, the primers used were: forward primer (CSo101), reverse primer (CSo104), 5′-ggaacaagaaggataatacc-3′. The primers CSo101 and CSo104 were used to prime sequencing reactions.

The first-round PCR mixture contained 1 μl of DNA in a total volume of 10 μl containing 200 nM concentrations of each primer constituted in 1× BioMix Red (Bioline, Boston, Massachusetts, United States) PCR reaction buffer. The second PCR mixture contained 1 μl of DNA in a total volume of 20 μl containing 200 nM of each primer in 1× reaction buffer. PCR amplifications were performed in a MJ Research DYAD 96-well thermocycler with the following cycling conditions: 95 °C for 4 min, followed by 40 cycles of 94 °C (1 min), 50 °C (1 min) and 72 °C (1 min) and final extension at 72 °C for 4 min. Second-round PCR products were monitored on a 2.0% agarose gel in 1× TAE buffer to check the quality, size and yield of the PCR products before proceeding to product purification and sequencing.

After purification of PCR products using the QIAquick multiwell PCR purification protocol (Qiagen), sequencing reactions were performed following the BigDye Terminator version 3.1 cycle sequencing kit protocol (Applied Biosystems, Foster City, California, United States). In brief, the PCR-sequencing reaction mixture contained 2 μl purified product containing between 10 and 120 ng of PCR product together with 8.0 μl PCR master mix containing 2 μl of forward or reverse primer, 0.5 μl BigDye mix, 1.75 μl of sequencing buffer and purified water. The PCR amplification was performed in 96-well plates under the following conditions: 25 cycles of 96 °C for 0.30 min, ramp 1.0 °C/s to 50.0 °C, 50 °C for 0.15 min, ramp 1.0 °C/s to 60.0 °C, 60.0 °C for 4.00 min, ramp 1.0 °C/s to 96.0 °C.

The sequencing products were then precipitated using the ethanol-sodium acetate precipitation method and placed in the ABI 3730 capillary sequencing machine for sample electrophoresis.

### Sequence Analysis

The SeqMan software program (DNASTAR, Madison, Wisconsin, United States) was used to analyse the electropherogram sequences. The sequence of each PCR product was manually checked by at least two investigators and a consensus reached or the assay repeated. The forward and reverse electropherogram sequences were assembled into a contiguous sequence and the consensus file examined to decide whether it represented a single allele or mixed alleles. Contiguous sequences showing two or more electropherogram peaks in the same position (in which a clearly majority allele could not be defined) indicated mixed alleles.

The polymorphic variants at both regions of the CSP sequence (Th2R and Th3R) were scored using the 3D7/NF54 allele as reference. Eighty codons at positions 323–402 (numbered according to the 7G8 sequence [9]) were aligned for all isolates, and the data entered in an Excel worksheet. Two separate data files were created; one for the single and clear majority alleles and one for all isolates including mixed alleles. In the first data file, the amino acid in each specific position was scored as 1 if it was identical to the amino acid present at that position in the 3D7/NF54 sequence. Non-identical amino acids were scored as 0. In the second data file, the scoring was as following: 0 for non-vaccine type amino acid, 1 if only an amino acid identical to the vaccine type sequence was present, and 2 if the vaccine type allele was mixed with other allele(s).

### Genotyping of *msp-1, msp-2,* and *glurp* for Estimation of MOI

Nested PCR was used to discriminate alleles of *msp-1* block 2 and *msp-2* and the R11 repeat region of *glurp* as previously described [12].

Following electrophoresis, gels were double-scored independently by two investigators, and either consensus was reached or the assay repeated. The minimum number of genotypes at each locus was determined for each sample and the results entered in an Excel worksheet. The overall genotypic complexity of each isolate was taken as the highest of the three numbers calculated at each locus. The mean of this minimum number was then calculated for each group, and termed the “multiplicity of infection” (MOI). This must be regarded as a minimum estimate of the true mean MOI [12].

### Blinding

All DNA extractions and laboratory analyses were performed by individuals blinded to vaccine assignment.

### Statistical Methods

An analysis plan was agreed upon prior to decoding the dataset into treatment groups. The endpoints for assessing the strain specificity of the vaccine were: 1) the relative proportion of the vaccine type allele, for each of the polymorphic amino acid sites at both T cell epitope regions (Th2R and Th3R), in the vaccine versus control group. The Fisher exact test (2-sided) was used to test the differences between RTS,S/AS02A and control group; and 2) the number of amino acids different from the vaccine type in each of the T cell epitope regions (Th2R and Th3R). The differences in the distribution of the number of amino acids different from 3D7/NF54 between the vaccine and the control groups were compared using a Mann–Whitney U test (2-sided Wilcoxon Rank Sum test). Statistical test results were not adjusted for multiple analyses.

To determine whether the vaccine affected the number of P. falciparum genotypes per infection, the frequency distribution of genotypes in vaccinated and control groups were compared using the Mann–Whitney U test as the primary outcome. Poisson regression was used to produce estimates of vaccine effect, adjusted for parasite density, age, and time to infection. However, the analysis did not produce an outcome that agreed with the non-parametric analysis, and therefore we concluded that the Poisson distribution did not adequately describe the data. An adjusted gamma regression was calculated and is presented as an exploratory (secondary) analysis, as this was able to fit the data more closely.

## RESULTS

### Participant Flow

Overall participant flow is summarized in Figure 1. The number of samples collected and used for sequencing is given in Table 1, and for estimation of MOI in Table 2.

### Numbers Analysed

The RTS,S/AS02A trial conducted in Mozambique enrolled two cohorts of children, one for estimation of vaccine efficacy against episodes of clinical malaria (Cohort 1), and the other for estimation of efficacy against infection (Cohort 2) [6]. In Cohort 1, 213 parasite-positive individuals were identified by the cross-sectional survey for parasite prevalence at study month 8.5. Of these, 82 were individuals vaccinated with RTS,S/AS02 and 131 had received control vaccines, representing 11.9% and 18.9% of participants surveyed in these two groups, respectively [6]. The *csp* genes of 208 (98%) of these were successfully amplified by PCR and sequenced. The electropherograms of 109 isolates (52.4%) indicated the presence of a single *csp* allele, eight (3.8%) indicated a single clear majority allele (with small background peaks indicating minority alleles at one or more of the polymorphic sites), and 91 (43.8%) gave mixed alleles. A total of 95 samples were analysed from 104 Cohort 1 participants admitted to hospital with clinical malaria for the first or only time: DNA from 90 of these (94.7%) was successfully amplified and sequenced. Forty-one (45.5%) of the PCR products gave electropherograms consistent with a single allele, 11 (12.3%) a single clear majority allele in the presence of small background peaks, and 38 (42.2%) were mixed alleles.

In Cohort 2, there were 323 children with first episodes of parasitaemia detected, but 82 of these children did not have a filter paper sample collected. The majority of these 82 samples corresponded to children who were febrile at the home visit and thus sent directly to the outpatient clinic where blood smear and microcapillary samples were taken, but filter paper was not. Thus DNA samples for genotyping analysis were available for 241 children with parasitaemia, and 223 (92.5%) of these samples were successfully amplified by PCR and the products sequenced. Of these, 157 (70.4%) contained a single allele, eight (3.6%) a single clear majority allele with small background peaks, and 58 (26%) gave mixed alleles. The observation of fewer mixed infections in Cohort 2 than in Cohort 1 may be at least partially explained by differences in both intervention and surveillance phases of the study. Children in Cohort 2 received anti-malarial treatment two weeks before the third dose of vaccine to clear parasitaemia prior to the start of the efficacy follow-up period, and underwent biweekly active detection and treatment of infection. Following up in Cohort 1 was by passive detection of malaria cases.

### Outcomes and Estimation

In each cohort we determined 1) the proportion of isolates with the CSP vaccine type (3D7) at each polymorphic amino acid position in the Th2R and Th3R epitopes in the vaccine and control groups; and 2) the number of amino acids different from vaccine type (3D7) within the Th2R and Th3R regions in the vaccine versus control group. There were eight and six amino acid positions in the Th2R and Th3R regions of the CSP sequence, respectively, which displayed polymorphism in our population and were therefore informative. Primary comparative analyses were performed using a conservative dataset comprising both mono-allelic isolates, and those isolates with a single clear majority allele against a background of one or more minor alleles. Mixed-genotype isolates were included in secondary comparative analyses.

Comparisons between groups were performed using Fisher's exact tests and by evaluating the 95% CI of the differences between groups. Post hoc, the study had the power to detect any between-group differences in the proportion of non-vaccine amino acid residues in the Th2R and Th3R epitope regions of at least 25% (cross-sectional survey in Cohort 1, conservative dataset) and of at least 20% for the other datasets.

**Figure 2:**
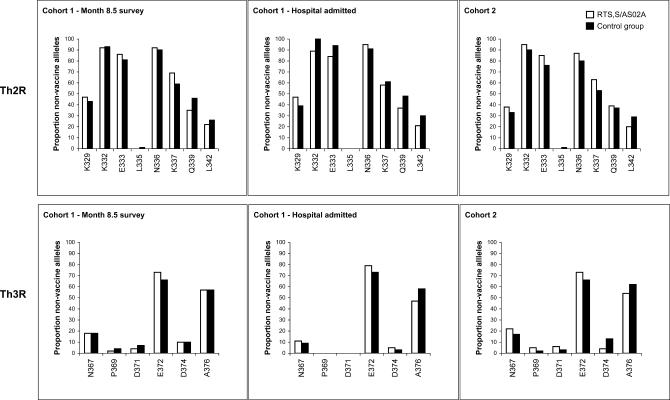
Percentage of the CSP Non-Vaccine Type Alleles for All Polymorphic Amino Acid Sites in the Th2R and Th3R Region in the RTS,S/AS02A Vaccine and Control Groups Denominators were, for Cohort 1, month 8.5 survey: *n* = 49, 68, in vaccine and control groups, respectively; for Cohort 1 hospital-admitted children: *n* = 19, 33; for Cohort 2: *n* = 79, 86.

The proportion of isolates that contained non-vaccine residues in the CSP Th2R and Th3R epitopes are shown in Figure 2. Among isolates collected at study month 8.5 from children in Cohort 1, primary analysis revealed no significant differences between vaccine and control groups at any amino acid positions in the Th2R region, nor at any position in the Th3R region. Similarly, no significant differences were found between treatment groups in the Cohort 1 participants admitted to hospital at any position in either epitope region. Among infections detected in Cohort 2, the vaccine-type residue 374-Asp in the Th3R epitope was replaced by Asn in 4% of the vaccine group infections, compared with 13% in the control group (difference in proportions 9%, 95% CI 0.8%–17.2%; *p* = 0.050), but there were no other significant differences between treatment groups in Cohort 2 at any position in either epitope region.

### Ancillary Analyses

In secondary analysis of Cohort 1 data at study month 8.5, including counts from mixed-allele infections, the vaccine-type Glu at residue 333 (Th2R) was replaced by Gln or Lys in 76% of vaccine group samples, compared with 64% of control samples (difference in proportions −12.5%, 95% CI −25% to 0.1%; *p* = 0.066), and the vaccine type Lys present at residue 337 in the Th2R region was replaced by Thr, Arg, or IIe in 52% of vaccine group samples compared with 38% of control samples (difference in proportions −14.7%, 95% CI −28.5% to −0.9%; *p* = 0.044). In Cohort 2, the vaccine-type residue 374-Asp in the Th3R epitope was replaced by Asn in 3% of the vaccine group infections when mixed alleles were included, compared with 9% in the control group (difference in proportions 6.6%, 95% CI 0.4%–12.7%; *p* = 0.054). Significant differences in substitution patterns between the control and vaccine groups were not seen at any other amino acid position in secondary analyses.

**Table 1:**
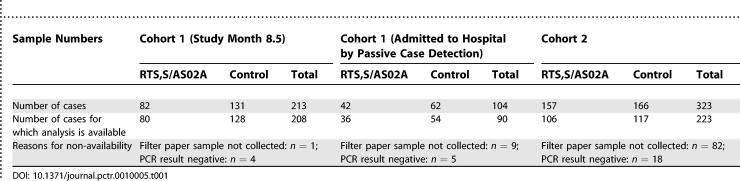
Overview of Data Used for Sequencing Analysis

**Table 2:**
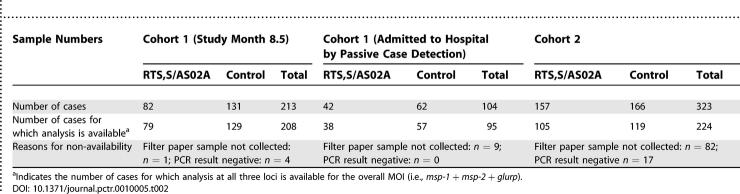
Overview of Data Used for MOI Analysis

**Figure 3:**
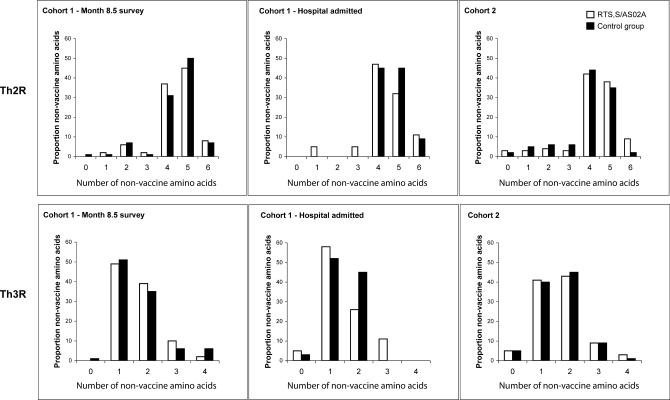
Proportion of Parasites with Given Numbers (*x*-axis) of Amino Acids Different from the Vaccine Type (3D7) in the Th2R and Th3R Region of CSP, in the Vaccine versus Control Groups Denominators were, for Cohort 1, month 8.5 survey: *n* =49, 68, in vaccine and control groups, respectively; for Cohort 1 hospital-admitted children: *n* = 19, 33; for Cohort 2: *n* = 79, 86.

We estimated the number of amino acids that differed from the vaccine type for each isolate. No significant differences were found between vaccine and control groups at either the Th2R or Th3R epitope regions in either cohort (Figure 3). Only three children (1.6%) from the control group and three children (2.0%) from the vaccine group harboured parasites with Th2R epitopes identical to those of the vaccine strain. Th3R epitope sequences identical to 3D7/NF54 were found in six children (3.2%) and five children (3.4%) in the control and vaccine groups, respectively. This contrasts to prevalences of 10% and 15%, respectively, among adult men in Gambia [11].

### Multiplicity of Infection

Parasite DNA from 208 samples from Cohort 1 (surveyed in study month 8.5), 95 from Cohort 1 children admitted to hospital, and 224 samples from Cohort 2 children, was typed successfully for *msp-1, msp-2,* and *glurp* genes and used for the analysis of MOI (Table 2).

**Figure 4:**
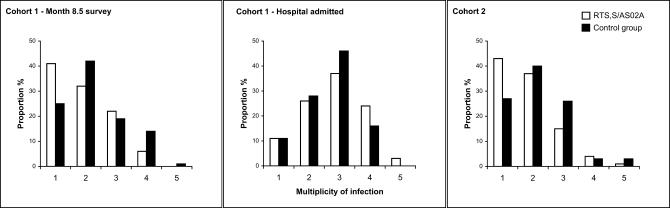
MOI in Emergent Infections for Both Vaccine and Control Groups Denominators were for Cohort 1, month 8.5 survey: *n* = 79, 129, in vaccine and control groups, respectively; for Cohort 1, hospital-admitted: *n* = 38, 57; for Cohort 2: *n* = 105, 119.

For Cohort 1 (study month 8.5) and Cohort 2, the overall minimum MOI (combining the data from the three loci) was significantly lower among infections in the vaccine group than among those in the control group (*p* = 0.035, *p* = 0.006, respectively, for the two cohorts*,* Wilcoxon rank sum test) (Figure 4). The mean MOI for Cohort 1 was 1.94 (sd 0.94) in the RTS,S/AS02A group and 2.24 (sd 1.01) among controls (proportional difference from gamma regression adjusted for parasite density 0.9; 95% C.I. 0.79–1.02; *p* = 0.098). Mean MOI for Cohort 2 was 1.83 (sd 0.89) and 2.16 (sd 0.97), respectively, for vaccine and control groups (proportional difference from gamma regression, adjusted for parasite density and time to infection 0.84; 95% CI 0.74–0.96; *p* = 0.007). Among Cohort 1 participants who were admitted to hospital, there was no significant difference between the vaccine (mean MOI 2.84, sd 1.08) and control groups (mean MOI 2.67, sd 0.87) in the genotypic complexity of infection analysis as indicated by the Wilcoxon test (*p* = 0.478) and the gamma regression adjusted for parasite density and disease severity (proportional difference 1.12; 95% CI 0.96–1.30; *p* = 0.154).

## DISCUSSION

Preventive measures against malaria disease and mortality are needed urgently as we enter the 21st century with a malaria burden among African children unchanged since the beginning of the previous century. The demonstration by Alonso et al*.* [6] that vaccination of children 1 to 4 years of age in Mozambique with GSK Biologicals candidate malaria vaccine RTS,S/AS02A protects against both infection and a range of clinical manifestations of malaria including severe disease, and that this protection is sustained over at least 18 months [7], is timely proof that development of an effective vaccine against malaria is feasible. The target antigen in RTS,S/AS02A, CSP of *P. falciparum,* includes regions that are highly polymorphic and may exist as dozens of variants within any given parasite population [8,9,11]. If the efficacy of RTS,S/AS02A is sequence-dependent, then protection may vary depending on the divergence of the CSP sequence in the predominant circulating strains. Theoretically, if a vaccine were widely deployed, sequence-dependent protection could select for genotypes at a parasite population level. We report here that among emergent P. falciparum infections in 222 vaccinated children as compared to 299 control individuals participating in the Mozambique trial of RTS,S/AS02A in 2003–2004 there was no evidence for selection of genotypes that differed in sequence from the vaccine type.

### Interpretation

The absence of measurable sequence-dependent selection in the Th2R and Th3R regions does not imply that a contribution of vaccine-induced cellular immune responses to the observed efficacy of RTS,S/AS02A can be ruled out. Indeed, there is ample evidence that humans vaccinated with RTS,S/AS02A generate immune responses to both epitopes [13,14], although a formal link between these responses and protection has yet to be made. CD4^+^ T cell responses to an invariant CSP epitope present in RTS,S have been associated with natural protection against infection [15], and recent genetic analysis of *csp* sequences encoding theTh2R and Th3R epitopes in 238 isolates from Thailand does not support the notion that diversity in these epitopes is generated in response to selection by sequence-specific human immunity in that population [16]. Furthermore, vaccination with RTS,S/AS02A may elicit T cell responses against the polymorphic region of CSP that recognises heterologous T cell epitopes, as both cross-clade recognition by HIV-specific T cells [17,18] and cross-reactivity of HPV-11 specific CD4^+^ T cells with other skin and genital HPV types [19] have been reported. Ongoing and future trials of RTS,S-based vaccines may shed light on the role of cell-mediated immune responses to conserved and polymorphic epitopes in the observed protection induced by the vaccine.

### Generalisability

Our analysis of sequence-dependent vaccine efficacy used direct-sequencing of DNA amplified directly from blood samples. All processes were performed in 96-well format from DNA extraction through to data collection. This innovative approach provides advantages over the dot-blot hybridisation method of Alloueche et al. [9,11] in being faster, easier to automate, and more robust, as the earlier method requires skill in interpretation of complex arrays of dot-blot data, and multiple reads by independent workers to eliminate observer error. The only limitation we encountered with direct sequencing was that a proportion of isolates were too complex at the nucleotide positions of interest in the Th2R- and Th3R-encoding regions of *csp*, due to the presence of multiple genotypes with different *csp* alleles, to unequivocally assign a full sequence. Thus, 43.8% of isolates from Cohort 1 and 26% from Cohort 2 were of mixed *csp* genotype with no clear majority allele, and did not contribute to our primary sequence analysis. This may potentially bias our findings if sequence-dependent selection occurs more commonly among these complex infections. This limitation also applies to dot-blotting methods, but could be addressed in future studies by cloning out and sequencing individual *csp* alleles from such mixed infections, adding substantially to the analysis effort.

An important parasitological parameter used in many clinical and epidemiological studies of malaria is the mean number of distinct parasite genotypes per infected individual, or MOI [12,20]. Studies of possible associations between MOI and risk of clinical malaria in Ghana, Mozambique, Tanzania, and PNG have been inconsistent [21–24], and as the relationship between MOI and malaria morbidity is possibly age-dependent [22], this is likely to vary considerably under different transmission conditions. Therefore, we must be cautious when interpreting our data about MOI. Indeed, evidence of a reduction in MOI was found in children vaccinated with the synthetic polypeptide vaccine SPf66 in Tanzania [25] and in Gambia [26], although the vaccine reduced risk of malaria in Tanzania [27] but not in Gambia [28]. Conversely, an increase in overall MOI was observed among Gambian men vaccinated with RTS,S/AS02A [11] who experienced a delayed time to first infection [4]. Among 527 emergent infections in the Mozambique trial, there was a reduction of MOI in RTS,S/AS02A-vaccinated children compared with controls both in Cohort 1 (at study month 8.5; *p* = 0.035) and in Cohort 2 (*p* = 0.006). No difference in MOI between RTS,S/AS02A-vaccinated children and control individuals was observed among Cohort 1 participants admitted to hospital with clinical malaria. The mechanism by which an efficacious vaccine might reduce MOI among emergent infections remains unclear, but in our analysis the reduction effect was shown to be independent of both parasite density and time to infection. A pre-erythrocytic vaccine such as RTS,S/AS02A may work in part by preventing or reducing emergence of parasites from the liver stage [3,5]. Our finding that there was no reduction in MOI in RTS,S/AS02-vaccinated children admitted to hospital with malaria may represent the emergence of new clones and is consistent with the hypothesis that new parasite types are more likely to cause illness than are those types already established in the host.

### Overall Evidence

We have found no evidence that the observed efficacy against malaria infection and morbidity of the RTS,S/AS02A vaccine among Mozambican children is sequence-dependent, and we have shown that fewer distinct parasite genotypes circulate in vaccinated hosts compared with controls. There has been no systematic review of genotypic multiplicity in the context of vaccination with RTS,S/AS02A. However, an analysis of a previous RTS,S vaccine trial conducted in Gambian adults [4] also revealed no differences in the prevalence of vaccine-type allele sequences among breakthrough infections between vaccine or control groups [11]. Moreover, although the vaccine reduced the incidence of infection in that trial, it did not reduce the MOI compared with controls. Although a genetic analysis of CSP T cell polymorphisms in individuals exposed to falciparum malaria along the Thai–Burmese border [16] indicated that naturally acquired immune pressure in the human host is unlikely to play any significant role in selecting and maintaining the extensive polymorphisms that occur naturally in the CSP Th2R and Th3R regions, such responses are typically weak and short-lived. This does not appear to be the case in the context of vaccination with RTS,S/AS02A [15,29], and the observation that vaccine-induced selection of escape mutants does not occur bodes well for candidate vaccines based on the CSP antigen.

These data were accrued in the context of a large Phase IIb trial. Nevertheless, should RTS,S/AS02A or similar pre-erythrocytic malaria vaccines be deployed on a large scale in the future, surveillance for evidence of sequence-dependent selection of breakthrough parasite genotypes would be a wise precaution. We have demonstrated that high-throughput direct sequencing of large numbers of field samples from parasite-positive individuals would be a feasible and robust approach to this end.

## SUPPORTING INFORMATION

CONSORT Checklist The trial protocol and CONSORT checklist list for the main trial have been previously published [6,7].
(49 KB DOC)

Trial Protocol (71 KB DOC)

## Abbreviations

CI: confidence interval
CSP: circumsporozoite protein
MOI: multiplicity of infection
sd: standard deviation
