# Supplementary material for: RTS,S/AS02A Malaria Vaccine Does Not Induce Parasite CSP T Cell Epitope Selection and Reduces Multiplicity of Infection
Source: PLoS Clin Trials. 2006 May 19;1(1):e5. doi: 10.1371/journal.pctr.0010005 (PMC1488895; doi:10.1371/journal.pctr.0010005)
Supplement: Trial Protocol — (71 KB DOC) [file pctr.0010005.sd002.doc]

## LSHTM GATES MALARIA PROGRAMME

**TITLE OF PROPOSAL**

Parasite genotyping for evaluation of protective efficacy of the malaria vaccine RTS, S/AS0A2 in children in Mozambique. *

**PRINCIPAL INVESTIGATOR(S)**

David Conway (LSHTM)

Carlota Dobaño (CISM, and Center for International Health, Hospital Clínic/UB)

Sonia Enosse (Instituto Nacional de Saúde-MOH; CISM, Mozambique),

Colin Sutherland (LSHTM)

**COLLABORATORS AND AFFILIATIONS**

Pedro Alonso (CISM, and Center for International Health, Hospital Clínic/UB)

Brian Greenwood (LSHTM)

Amanda Leach (GSKBio)

Jahit Sacarlal (CISM)

Ricardo Thompson (Instituto Nacional de Saúde-MOH, CISM)

Jessica Milman, (Malaria Vaccine Intiative)

**SUMMARY (200 words)**

The candidate malaria antigen RTS,S comprises a significant portion of the circumsporozoite protein (CSP) of *P. falciparum* linked to the hepatitis B surface antigen and expressed in yeast. This vaccine is currently being tested in a Phase IIb trial in Manhiça, Mozambique, in which children aged 1-4 year have been immunised with either three doses of RTS,S plus the GSK adjuvant AS02, or an alternative paediatric vaccine vaccine (Children > 24 months of age recieve hepatitis B vaccine; Children <24 months receive 7-valent pneumococcal vaccine at Doses 1 and 3 and *Haemophilus influenzae* type B vaccine at Dose 2). Two cohorts of children are being studied. Cohort 1 comprises approximately 1600 children in whom the main trial end-points will be the incidence of clinical attacks of malaria and the prevalence of anaemia. Cohort 2 comprises 400 children in whom the main trial end-point will be time to first *P.falciparum* infection.

The proposed study will use molecular genetic typing methods to determine two important parasitological parameters **a)** if there is detectable strain-specificity in the effect of the vaccine **b)** if vaccination alters the multiplicity of subsequent infections. Both these parameters will be examined in both trial cohorts.

***** The proposed work is ancillary to the current double-blind randomised controlled phase IIb study to evaluate the safety, immunogenicity and efficacy of GlaxoSmithKline Biologicals' candidate malaria vaccine RTS,S/AS02A, administered IM according to a 0, 1 and 2 month vaccination schedule in toddlers and children aged 1 to 4 years in a malaria-endemic region of Mozambique (GSK study id: 257049/026 (Malaria-026); registered as **NCT00197041**). This is a collaborative study of GSKBio, the Malaria Vaccine Initiative (MVI), CISM Mozambique and the University of Barcelona. The trial is currently being conducted under US IND.

**(a) Background and Justification**

Malaria continues to be a major cause of morbidity and mortality in many countries of Africa, especially in Sub-Saharan Africa (Greenwood & Mutabingwa 2002). The efforts to control malaria have mainly focused on prompt and effective treatment of confirmed or suspected cases and the use of insecticides to control the vector. However, the malaria problem has increased in recent years with the lack of effective vector control (partly as a result of resistance of malaria vectors to insecticides), the emergence of multi-drug resistant *P. falciparum* parasites and the weak quality and performance of the health services in affected countries (Hastings & D'Alessandro 2000). Whereas better use of available control measures and the search for new, cheap and effective drugs are the main aims of current efforts in malaria research, sustainable control of malaria in the long-term can best be achieved when highly effective vaccines can be used in combination with other control measures.

Several malaria vaccine candidates have been tested in trials in malaria endemic countries (Graves & Gelband 2003), including the pre-erythrocyte vaccine RTS,S (Gordon *et al.*, 1995), a recombinant polypeptide that contains most of the circumsporozoite protein (CSP) sequence of *Plasmodium falciparum* fused to Hepatitis B virus surface antigen (Stoute *et al*. 1997). In a trial conducted in the Gambia in semi-immune adults, RTS,S administration with the adjuvant AS02A was demonstrated to be safe and to provide significant protection against *P. falciparum* infection (Bojang *et al.* 2001).

The CSP is the predominant protein found in the surface of the sporozoite. Studies of the genetic diversity of the gene encoding the CSP of *P. falciparum* have demonstrated the existence of high levels of genetic polymorphisms in isolates from different areas in Africa (Escalante, *et al.* 2002). This high number of alleles should be considered when testing the efficacy of CSP-based vaccines. In the Gambia, the RTS,S/AS02A vaccine was demonstrated to induce protection against first *P. falciparum* infections that was not strain-specific. The frequency of the vaccine allele-type, which was derived from the sequence of the CSP allele in laboratory clone 3D7, was similar to that of all other *csp* alleles studied in the vaccine vs control group (Alloueche *et al*. 2003). The key polymorphic sites in the *csp* gene which are encompassed by the RTS,S antigen are the T-cell epitopes at the carboxy-terminus of the protein, designated Th2R and Th3R. Polymorphisms in these epitopes were the focus of the evaluation of strain-specificity of vaccine efficacy in the Gambian trial (Alloueche *et al*., 2003). Evidence was also found in this study that RTS,S modified the average number of clones (multiplicity) carried during post-vaccination parasitaemia. Modification of multiplicity of *P. falciparum* infections was also found after vaccination with the chimaeric peptide multimer vaccine SPf66 (Haywood *et al.*, 1999).

To evaluate strain-specific vaccine effects in a trial evaluating the efficacy of the RTS,S/AS02A vaccine in Mozambican children, finger-prick blood samples will be used for sequence analysis of the Th2R and Th3R sequences in the *csp* gene of slide-positive individuals in both vaccine and control arms of the trial. Vaccine effects on multiplicity will be investigated using polymorphic antigens loci *msp1*, *msp2* and *glurp* to measure multiplicity in parasitaemic individuals from both vaccine and control arms of the study.

**(b) OBJECTIVES**

The objectives of the study are:

1) To investigate whether vaccination with RTS,S/AS0A2 modifies the frequency of the vaccine type *csp* allele (th2r, th3r sequence regions) in subsequent incident *P. falciparum* infections:

2) To investigate whether the malaria vaccine RTS,S/AS0A2 modifies the number of *P. falciparum* genotypes (multiplicity of infection) present in subsequent infections:

Both objectives will be pursued using three sources of parasite DNA:

- From first microscopy-confirmed infection identified by ACD (cohort 2)
- from microscopy-confirmed infections identified in the cross-sectional survey of all cohort 1 participants 6.5 months after the third vaccination.
- In cohort 1 participants with microscopy-confirmed clinical malaria identified by PCD and admitted to the hospital.

**(c) STUDY DESIGN AND METHODS**

**1. Study area, population and vaccine trial**

Samples for molecular analysis will be obtained during the course of a vaccine trial that has been carried out in Manhiça district, Southern Mozambique. Details of the study area and population have been previously described (Alonso *et al*. 2001). Malaria transmission in the area is perennial with seasonal variation and the majority of cases occur during the rainy season. The entomological inoculation rate in the region is estimated at 15 infectious bites per person per year (Saúte *et al.* 2003), but clinical attack rates suggest a higher level of transmission (Saute *et al.,* unpublished).

*Vaccine and Trial design*

The vaccine under evaluation in Mozambique is the well characterised vaccine RTS,S/AS02A produced by Glaxo SmithKline Biologicals (GSKBio) (Stoute *et al*. 1997, Kester *et al*. 2001) . RTS, S/AS02A is a recombinant protein malaria vaccine that contains a large portion of the circumsporozoite protein (CSP) sequence of *P. falciparum* fused to the hepatitis B virus surface antigen.

The trial (Malaria-026) is a double-blind randomised controlled design that has enrolled approximately 2000 children aged 1 – 4 years (inclusive). This trial uses a two-cohort design to examine the efficacy of the vaccine at two points in the pathogenesis of malaria disease: infection and clinical disease of moderate severity. In this current proposal we will analyse parasite DNA from these major end-point samples.

Cohort One – Efficacy against clinical malaria disease

The primary objective of the trial is to determine vaccine efficacy against clinical malaria disease. One thousand six hundred and five children have been enrolled to Cohort 1. The primary endpoint is the time to the first clinical episode of symptomatic *P falciparum* malaria over a 6 month observation period starting 14 days after dose 3. A case is defined as the presence of *P. falciparum* asexual parasitaemia above 2500 per µl on Giemsa stained thick blood films AND the presence of fever (axillary temperature ≥ 37.5°C) at the time of presentation AND occurring in a child who is unwell and brought for treatment to a healthcare facility. There is no parasite clearance with drug treatment in children enrolled to this cohort.

Cohort Two – Efficacy against infection

Four hundred and seventeen have been enrolled to Cohort 2. The main endpoint of this cohort is the time to first malaria infection, which is defined as the first recording of infection of asexual stage falciparum parasites detected by the active detection of infection surveillance or passive case detection, over a period starting 14 days after last vaccination and until 6½ months after last vaccination.

Four weeks prior to the start of surveillance for malaria infection (i.e. 2 weeks before the third vaccination), asymptomatic parasitaemia will be cleared in children enrolled into Cohort 2 with amodiaquine-SP (sulfadoxine and pyrimethamine : 25 mg/kg body weight p.o. and 1.25 mg/kg body weight p.o. single dose; amodiaquine: 10 mg/kg body weight p.o. once daily for three days). The prevalence of asymptomatic parasitaemia in the study area is high, therefore all children will receive a drug for clearance of malaria parasites without prior blood sampling to determine parasitaemia. The absence of parasitaemia will be checked by taking blood for a blood slide on the day of administration of Dose 3. Any child with any level of parasitaemia at this point will be treated with a second-line treatment for malaria and will not undergo the surveillance period for active detection of infection. All such children will not contribute to the endpoint for vaccine efficacy in Cohort 2.

The surveillance period for malaria infection will begin 14 days after the third dose of vaccine (day 74). Surveillance visits will occur every two weeks for the following 2½ months (six visits) and then monthly for 3 months. Blood samples will be taken for examination of malaria parasitaemia and parasite genotyping at each visit. At first detection of malaria infection with asexual forms of *P. falciparum* in a child, either by field worker surveillance or on clinical presentation with malaria disease at a health facility, the child will be considered to have met the endpoint for vaccine efficacy in Cohort 2. At this point the child will be treated and no further visits for surveillance of infection will be undertaken until the last visit at 6 ½ months post Dose 3 when all children will be sampled.

**2. Sample size determination**

**Cohort 1**

There are 213 parasite positive samples from cohort 1 detected at the final cross-sectional parasitological survey.

There are 102 frozen erythrocyte pellets from cohort 1 participants admitted to the hospital with clinical malaria identified by PCD during the surveillance period.

**Cohort 2**

There are 241 children in cohort 2 identified as parasite-positive by ACD, with blood samples collected on filter-paper, and a further 11frozen erythrocyte pellets from Cohort 2 participants who presented to the clinic as symptomatic and were treated for clinical malaria as in-patients.

Assuming these are evenly distributed between vaccine and placebo groups, we are likely to have a power of between 17 and 62% to detect a difference in prevalence caused by selection against the vaccine strain at either locus, when analysed at each polymorphic position sequentially, and assuming a selection coefficient s=0.5.

**3. Laboratory methods**

All laboratory work will be performed by individuals blinded to vaccine assignment. Vaccine code will assigned after completion of analyses.

*DNA extraction from filter paper and frozen erythrocyte pellets*

The filter paper blood samples are air dried, individually wrapped in aluminium foil, placed in a box containing desiccant (silica gel) and stored at 4^o^C until use. All samples are labelled with a sample identification number.

DNA extraction from filter paper will be performed as previously described (Plowe *et al.* 1995). In brief a half of blood spots collected onto filter paper will be incubated with 1 ml of 0.5% saponin in phosphate buffer saline (PBS, pH 7.0) overnight at room temperature. After centrifugation the saponin-PBS solution is removed and the samples washed twice with PBS. The PBS is aspired and 100 µl of distilled H_2_0 plus 50 µl of 20% chelex®-100 resin (Bio-Rad Laboratories) are added. The samples will be heated at 100 ºC for 8 minutes, centrifuged at maximum speed for 5 min. DNA samples will be used immediately or stored at –20 ºC for later amplification. An alternative method using QIAGEN blood DNA extraction kits will be evaluated on a pilot study with 50 blood-spot samples, and if this method offers significant improvement it will be adopted. All frozen blood extractions will be performed using the QIAGEN procedure.

*Determination of csp polymorphic sequences*

The distribution of polymorphic variants within the Th2R and Th3R regions of *P falciparum* CSP will be determined using high-throughput direct sequencing of PCR products. The relevant portion of the gene will be amplified and a sequence strategy devised and validated in a pilot study of 50 blood spots. The sequencing will be performed using an ABI 3730 capillary sequencer (provided by the GMP). The sequence of each PCR product will be manually checked by at least two investigators and a consensus reached or the assay repeated. For those isolates in which a clear majority allele is not determined from the sequence electropherogram, the majority allele will be determined by selected use of the PCR-single-stranded oligonucleotide probing (SSOP) technique as previously described (Alloueche *et al*. 2000). Any remaining ambiguities will be resolved by cloning of PCR products and sequencing.

*Genotyping of msp-1, msp-2 and Glurp and detection of multiplicity of infection*

Allele specific, nested PCR will be used to detect *msp1* block 2 and *msp*2 allelic families and allelic variants present within the R11 repeat region of the blood stage antigen *glurp* as described by Snounou *et al.* (1999). Following electrophoresis, staining with ethidium bromide and observation under ultra-violet illumination, bands corresponding to different parasite allelic forms will be distinguished and counted and the number of genotypes for *msp* 1*, msp* 2 and *glurp* loci determined. Gels will be double-scored independently by 2 investigators, and either consensus reached or the assay repeated.

**4. Data processing and statistical analyses**

The cases contributing to the endpoints for infection in cohort 2 as defined above (section 1), and parasite-positive individuals identified in the cross-sectional survey 8 months after vaccination in both cohorts will be provided from the final trial database of Malaria-026 by GSK. After completion of all laboratory analysis, the vaccine assignment will be applied to the final database of this study by GSK.

The main endpoint for assessing the strain specificity of the vaccine will be the relative proportion of the vaccine type allele in the vaccine vs. control group. For each infection, the predominant allele will be counted in the analysis. If two alleles are equally abundant in an infection, two analyses will be performed. In the first, these samples will be excluded. In the second analysis those mixtures including the vaccine-type allele will be included with the vaccine type. Proportions in the vaccine vs. control groups will be compared using the chi-square test.

Analysis on the multiplicity of infection will be done as described in the analyses of previous vaccine trials (Alloueche *et al* 2003, Haywood *et al* 1999). Briefly, to determine whether the vaccine reduces the number of *P. falciparum* genotypes, the mean numbers of genotypes in each allelic family of *msp1*, *msp2* and *glurp* will be computed and the differences between means in the vaccinated vs. control groups compared using the Wilcoxon test. Regression analysis will be used to assess the effect of the vaccine on multiplicity of infection adjusted for geometric mean parasite density, disease severity and age. All data produced will be entered by study identification number and provided to the main trial data manager.

A detailed Reprt Analysis Plan will be agreed upon by all investigators before the analysis is performed.

**(e) REFERENCES**

Alloueche A, Milligan P, Conway D, *et al*. (2003) Protective efficacy of the RTS,S/AS02 *Plasmodium falciparum* malaria vaccine is not strain specific. *American Journal of tropical medicine and hygiene* **68:** 97-101.

Alloueche et al. (2000) High-throughput sequence typing of T-cell epitope polymorphisms in Plasmodium falciparum circumsporozoite protein. Molecular and Biochemical Parasitology **106**: 273-282

Alonso PL, Saute F, Aponte JJ *et al* (2001) Manhiça DSS, Mozambique. In: *Population, Health and Survival at INDEPTH Sites.* Vol. 1, chapter 15 (eds International Development Research Center) Ottawa, pp 189-195

Bojang KA, Milligan PJM, Pinder M *et al*. (2001) Efficacy of RTS,S/AS02 malaria vaccine against *Plasmodium falciparum* infection in semi-immune adult men in the Gambia: a randomized trial. *The Lancet* **358** (8), 1927-34.

[Gordon DM, McGovern TW, Krzych U, et al. (1995)](http://www.ncbi.nlm.nih.gov:80/entrez/query.fcgi?cmd=Retrieve&db=PubMed&list_uids=7769295&dopt=Abstract) Safety, immunogenicity, and efficacy of a recombinantly produced Plasmodium falciparum circumsporozoite protein-hepatitis B surface antigen subunit vaccine.
*J Infect Dis*. **171:** 1576-85.

Graves P and Gelband H (2003) Vaccines for preventing malaria. Cochrane Database Syst Review 2003;(1):CD000129.

Greenwood BM and Mutabingwa TK (2002) Malaria in 2002. Nature **415**, 670-672

Hastings IM & D'Alessandro U (2000) Modelling a predictable disaster: the rise and spread of drug-resistant malaria. *Parasitol Today* 16 (8), 340-347

Haywood M, Conway DJ, Weiss H, *et al.* (1999) Reduction in the mean number of *Plasmodium falciparum* genotypes in Gambian children immunized with the malaria vaccine SPf66. *Transactions of the Royal Society of Tropical Medicine and Hygiene* **93** (1), 65-68

Kester KE, McKinney DA, Torniieporth N, et *al*. (2001) Efficacy of recombinant circumsporozoite protein vaccine regimens against experimental *Plasmodium falciparum* malaria. Journal of Infectious Disease **183**, 640-647

Plowe CV, Djimde A, Bouare M, Doumbo O & Wellems TE (1995) Pyrimethamine and Proguanil resistance-conferring mutations in Plasmodium falciparum dihydrofolate reductase: Polymerase chain reaction methods for surveillance in Africa. *American Journal of tropical medicine and hygiene* **52**(6), 565-568

Saúte F, Aponte J, Almeda J *et al*. (2003) Malaria in Southern Mozambique: incidence of clinical malaria in children living in a rural community in Manhiça district. *Transactions of the Royal Society of Tropical Medicine and Hygiene*, in press 000.000.

Snounou G, Zhu X, Siripoon N, *et al*. (1999) Biased distribution of msp1 and msp2 allelic variants in *Plasmodium falciparum* populations in Thailand. *Transactions of the Royal Society of Tropical Medicine and Hygiene* **93** (4), 369-374

Stoute JA, Slaoui M, Heppner G, *et al*. (1997) A preliminary evaluation of a recombinant circunsporozoite protein vaccine against *Plasmodium falciparum* malaria. *N Engl J Med* **336**, 701-707

**(d) TIMETABLE AND MILESTONES**

| **Period** | **Activities** |
| --- | --- |
| January 2004 | Submit protocol to GMP |
| January to March 2004 | Colin Sutherland to develop and validate all laboratory procedures using non-trial samples from Manhiça. SOPs written. |
| May to July 2004 | Sonia Enosse to perform DNA extractions and initiate multiplicity of infection typing in Manhiça. Training of local laboratory technicians employed by the project |
| July 2004 | Colin Sutherland and David Conway to visit Manhiça to review laboratory and analytical procedures.  Data analysis plan developed via email and/or telecom. |
| July to December 2004 | Carlota Dobaño to supervise continuation of multiplicity of infection typing in Manhiça.  Sonia Enosse, Colin Sutherland and David Conway to generate *csp* sequence data at LSHTM |
| April – May 2005 | Report analysis plan to be drawn up by John Aponte and Marc Lievens. To be circulated and agreed prior to decoding the data and final analysis. |
| June 2005 | Analysis and writing.  Colin Sutherland, David Conway, Brian Greenwood, Jessica Milman, Amanda Leach, Ripley Ballou and Sonia Enosse to meet with Pedro Alonso and Carlota Dobaño in Barcelona to discuss the results (date to be confirmed). |
